# Development and Validation of a Sexual-Outlook Questionnaire (SOQ) for Adult Populations in the Republic of Korea

**DOI:** 10.3390/ijerph17228681

**Published:** 2020-11-23

**Authors:** Sun Houng Kim, Hyang Yuol Lee, Seung Young Lee, Bum Suk Lee

**Affiliations:** 1 National Rehabilitation Center, Seoul 01022, Korea; luciakim@korea.kr (S.H.K.); iambs@korea.kr (B.S.L.); 2 College of Nursing, The Catholic University of Korea, Seoul 06591, Korea; 3 College of Nursing Science, Kyung Hee University, Seoul 02447, Korea; noahmam@naver.com

**Keywords:** sexual health, surveys and questionnaires, population health

## Abstract

A Sexual Outlook Questionnaire (SOQ) that can apply to a wide range of Korean populations, including disabled people, was necessary for comprehensive research on improving clinical practice of sexual education and developing sex-related intervention programs. We developed the SOQ and tested its validity with exploratory and confirmatory factor analysis and multi-trait/-item matrix analyses. Internal consistency was assessed using Cronbach’s α coefficient for item total correlations. We studied a total of 334 married or previously married adults with no cognitive impairment in the community settings. The eleven survey items were included in the final SOQ. Three factors were found: The first, “personal benefit”, was devoted to the impact of one’s sexual life and included four questions about the health-promoting effects and their recognition of healthiness, youth, and vitality as benefits of their sexual life. The second, “relational value”, included four questions about sex as an expression of love and means of communication, and its effect on the improvement of their relationship with their spouse (partner). The third, “sexual endeavor”, included three questions about the handling of sex-related problems, consulting with an expert, and sexual education. The questionnaire can briefly measure the sexual outlook of any married or previously married adult, regardless of disability.

## 1. Introduction

Sexual outlook refers to individuals’ values and convictions toward sex, including systematic and consistent thoughts, emotions, views, patterns of behavior, and knowledge, often affected by the culture to which the individuals belong [1]. Sexual outlook incorporates physical and mental satisfaction, the meaning of sex, the handling of sexual matters, the diversity of one’s sexual life, the use of resources such as sexual counseling and education, prejudice toward sex, knowledge about sex, openness to sex, and sexual responsibilities [2,3,4,5]. In other words, people’s sexual outlooks reflect their education, environment, personal experience, and sociocultural elements. People express their sexual behaviors and thoughts based on their values and beliefs about sex. However, sexual and reproductive health are not discussed or are insufficiently discussed in many countries because issues of sexuality make people feel uncomfortable [6].

Sexual activities positively affect physical and mental health [7]. A positive and open outlook towards sex aligns with a higher sexual need, more knowledge about sex, and more sexual activities [8]. As knowledge and outlooks toward sex closely relate, better sexual knowledge aligns with the formation of good sexual outlooks that enable adolescents, adults, and elders to maintain regular sexual activity [9]. To remain satisfied in one’s sexual life, it is important to have positive and open outlooks toward sexual and reproductive health that can improve one’s quality of life, regardless of disabilities.

Sexual outlook researchers in Korea used modified instruments to consider current social or cultural situations [10]. In using any instrument in a culture and environment outside the area in which the instrument was developed, a validity confirmation study must be performed [5]; in Korea, the use of instruments is limited to previously developed instruments to measure sexual outlook that were not validated with research [3,4,11]. A strong need exists to develop and validate a new universal instrument that briefly measures Korean people’s sexual outlooks, reflecting various population demographic characteristics.

The purpose of the present study was to develop the Sexual Outlook Questionnaire (SOQ) that can apply to a wide range of Korean people, including those with disabilities, and to validate the best set of possible items for the comprehensive use of research in improving clinical practice of sex education and developing sex-related intervention programs to protect the human rights of general adult populations.

## 2. Materials and Methods

### 2.1. Study Design

This was a methodological study to develop and evaluate the best set of SOQ items for Korean adults and to test its validity and reliability.

### 2.2. Study Participants

Participants in the present study were married or previously married adults of at least 19 years of age residing in the local society with no cognitive impairment. Participants who were disabled were outpatients or participating in health examinations whose disability had occurred at least one year before and who consented to participate in the present study. Those not eligible for the present study were people less than 19 years of age who were never married, with cognitive impairment, hospitalized for treatment, or whose disability had occurred within less than one year, and who did not consent to participate in the present study.

### 2.3. Procedures

A convenient sampling was used in Seoul and the Gyeonggi-do area. The number of participants in the present study was 350 people with and without disabilities (130 with disabilities and 220 people without) residing in Seoul and the Gyeonggi-do regions. The number of participants was decided according to the argument that the ratio of cases and measurement variables should be more than 10:1 in an exploratory factor analysis [12] and the appropriate number of participants is between 200 and 400 for researchers to construct a structural equation model for confirmatory factor analysis [13,14]. We excluded data from 16 respondents who gave the same answer to all questions or who responded insincerely; thus, the data from a total of 334 respondents were used in the analysis.

### 2.4. Patient and Public Involvement

The SOQ for Korean adults used in the present study was developed and its validity was tested in two stages: the questionnaire-development stage and the questionnaire test stage. The literature related to sexual health was surveyed and in-depth interviews were conducted in the questionnaire development stage with those who were sexually active, including two people with myelopathy, one couple where one spouse had a stroke and the other had no disability, and three people without disabilities to determine the primary preliminary questionnaire. In the second step, we conducted a Delphi survey with seven experts, including physicians in rehabilitation medicine and gynecology and a clinical psychologist with more than five years of clinical practice, a Ph.D. in social welfare, a Ph.D. in nursing, a sex educator, and a Ph.D. in counseling to verify and revise the content validity of the primary preliminary questionnaire. A secondary Delphi survey was conducted with five experts in sex counseling to complete the secondary preliminary questionnaire. We conducted a preliminary survey using the secondary preliminary questionnaire with 10 people with disabilities and 10 without disabilities to check the appropriateness of the wording, the arrangement of the questions, and the applicability of the questions. After consulting with a scholar in Korean literature, we confirmed the final version of the questionnaire.

### 2.5. Questionnaire Development

Based on the literature review, we collected questions related to sexual outlook characteristics. In-depth interviews were conducted to extract meaningful statements about participants’ sexual lives. Interviews began with semi-structured questions such as, “What do you think about sex?” “How is your entire life affected by your sexual life?” “What is your wish for your sexual life in your future?” More detailed questions followed. We classified the questions extracted from the literature review and the interviews by similarity, segregated into five categories: values of sex (6 items), benefits of sex (9 items), meanings of sex (6 items), sexual assertiveness (6 items), and sexual responsibility (4 items). Therefore, the primary preliminary questionnaire consisted of 31 questions. Sexual outlook was measured on a 5-point Likert-type scale including the options of “strongly disagree”, “disagree”, “so-so”, “agree”, and “strongly agree”. A higher score indicated a positive sexual outlook.

The initial questionnaire included preliminary items of 29 questions measuring sexual outlooks with 10 questions about the participants’ general characteristics and the 10-point visual-analog scale to express the importance of sexual activity in one’s life. General characteristics included gender, age, education level, marriage status, employment, religion, disease history, presence of disability, type of disability, and time of disability occurrence.

### 2.6. Data Collection

The study proposal was approved by the Institutional Review Board of the National Rehabilitation Center (Approval No. NRC-2017-02-013). The data accrued from July to October 2017. Four research assistants who had research experience helped the data collection. They received about 30 min of education on the purpose of the study, the participants, and the method of collecting the questionnaires. Researchers explained the purposes and procedures of the study to participants and informed participants that their confidentiality would be secure, they could withdraw from the study any time during the survey, and their participation was voluntary. Participants completed the questionnaire on their own, but research assistants helped those who needed help due to a disability. The completed questionnaire was collected and sealed with a separate consent. A small gift was provided to each participant. The time taken by participants to complete the questionnaire was about 15 to 20 min.

### 2.7. Data Analysis

The SOQ’s validity and reliability were examined using IBM SPSS Statistic 22.0 and AMOS 22.0 software programs. The descriptive statistics and frequency analyses were performed with respect to the participants’ general characteristics and study variables. The content validity was tested by measuring the questions’ appropriateness on a 4-point scale. The content validity index (CVI) was calculated and questions having a CVI of 0.80 or higher were selected. To test construct validity, x Pearson’s correlation of the questions was analyzed and an exploratory factor analysis and confirmatory factor analyses were performed. We performed the Kaiser–Mayer–Olkin (KMO) test, Bartlett’s sphericity test, and the Measure of Sampling Adequacy (MSA) test to determine if the data were appropriate for the factor analysis. For the exploratory factor analysis, a principal component analysis (PCA) and a varimax rotation method were used to determine the number of factors with an eigenvalue of 1 or higher and which factor loadings were 0.5 or higher [12]. After determining the number of factors, a confirmatory factor analysis was utilized to calculate model fitness. The fitness of the structural equation model was determined by considering the χ^2^ value, the degree of freedom (df), the root-mean-square residual (RMR), the root-mean-square error of approximation (RMSEA), the goodness-of-fit index (GFI), the comparative fit index (CFI), and the Tucker–Lewis Index (TLI) [14]. A multi-trait/-item matrix analysis was performed to test convergent and predictive validity, and Cronbach’s α coefficient evaluated the reliability of the developed questionnaire.

## 3. Results

### 3.1. General Characteristics

The total number of participants was 334, and their average age was 48.26 ± 11.09 years. The greatest number of participants were in their 40s, with men comprising 174 (52.1%) of participants. Most participants (232, 69.5%) were employed. About two-thirds of participants (210, 62.9%) resided in Seoul, with the rest residing in Gyeonggi-do. A quarter of participants (92, 27.5%) had a chronic disease. The number of the participants with a disability was 121 (36.2%). Of the total 334 subjects, 115 subjects (34.4%) responded that they had a problem in their sexual life (see Table 1).

### 3.2. Possible Items and Content Validity

We tested the content validity of the primary preliminary questionnaire with the seven experts regarding the questionnaire’s appropriateness on a 4-point scale. We removed two questions in which the CVI was 0.80 or lower and revised eight other questions. As a result, we prepared the secondary preliminary questionnaire, including 29 questions. We conducted a secondary Delphi survey on the selected 29 preliminary questions with five sexual counseling experts and did not remove any question for which the CVI was 0.80 or higher and revised two questions. With the remaining 29 questions, we conducted a preliminary survey with 10 people with disabilities and 10 people without disabilities to investigate their understanding of the words and the applicability of the questionnaire. We prepared the final version of the questionnaire after consulting with a scholar of Korean literature about the sentences and wording, and revised four questions accordingly.

### 3.3. Construct Validity

#### 3.3.1. Exploratory Factor Analysis

Before the factor analysis, we verified the presence of the common factors suitable for the factor analysis because the KMO value was 0.865 and the result of the Bartlett’s sphericity test was significant (χ^2^ = 1996.341, *p* < 0.001). We determined the number of factors in the preliminary questionnaire according to Kaiser’s rule that the eigenvalues should be 1 or higher and the recommendation that the cumulative explained variance should be 60% or higher [13,14].

We removed six questions (x4, x6, x7, x8, x9, x10) where the factor loading value was 0.50 or less [12]. In addition, we removed 12 questions (x3, x5, x6, x13, x16, x21, x22, x23, x24, x25, x26) where the factor loading value was 0.35 or higher on two or more subfactors to clearly differentiate the structure between factors. Two questions formed as a factor were removed because each factor must have at least three items [14]. As a result, we extracted a total of 11 questions corresponding to the final three subfactors. The communality of the individual questions ranged from 0.613 to 0.832. The three extracted subfactors explained 73.477% of the total variance, conforming to the recommendation that the cumulative explained variance should be 60% or higher [14,15]. The correlation coefficients of the factor loading values of the individual questions (pattern matrix) found by the factor analysis with the question scores ranged from 0.465 to 0.701 (see Table 2).

The names of the three subfactors extracted by the factor analysis mostly corresponded with the concepts of the subfactors proposed in the study’s early stage.

The question that showed a high loading for Factor 1 was “Sexual life makes me feel alive”. As Factor 1 consisted of questions related to an increase in vitality and the promotion of health, it was named “personal benefit of sex”. The question that showed the highest loading to Factor 2 was “Sexual life improves the relationship with the spouse (partner)”. Factor 2 consisted of questions related to the promotion of partnership, including improvement of relationship, expression of love, methods of communication, and discussion on sexual matters. As the questions were about the importance of a sexual relationship, Factor 2 was named “relational value of sex”, in line with the theoretical framework.

The question that showed a high loading for Factor 3 was, “If I have a sex problem, I will consult with an expert”. As Factor 3 consisted of questions related to sex education and the use of sexual aids, representing assertive sexual actions, it was named “sexual endeavor”. (see Table 2).

#### 3.3.2. Confirmatory Factor Analysis

We performed a confirmatory factor analysis using the structural equation model to calculate model fitness of the final version of the questionnaire. The results showed that χ^2^ = 113.417(*p* < 0.001) and χ^2^/df = 2.766, indicating χ^2^/df was less than 3. The results also showed that the RMR = 0.047 (<0.05), the RMSEA = 0.073 (<0.10), the GFI = 0.941 (>0.90), the CFI = 0.963 (>0.90), and the TLI = 0.950 (>0.90), indicating that all criteria were satisfied. Therefore, the fitness of the questionnaire developed in the present study was verified (see Table 3).

**Figure 1:**
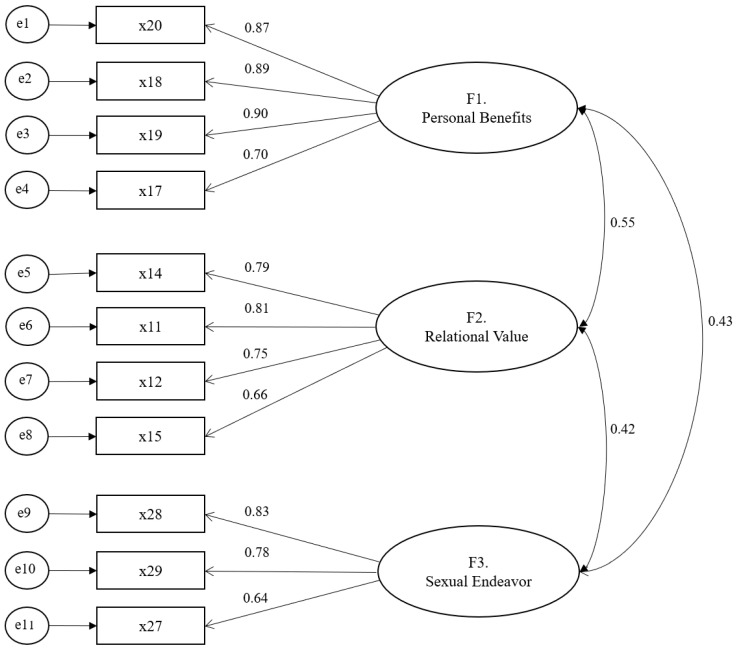
Confirmatory Factor Analysis of 11-Item SOQ.

The correlation coefficients between the subfactors in the model ranged from 0.42 to 0.55 (see Figure 1; Supplementary material Figure S1). The standardized coefficient (β) of the measurement variables of the individual subfactors ranged from 0.64 to 0.90, which was higher than 0.50. We calculated the construct reliability (CR) and the average variance extracted (AVE) to analyze the reliability of the subfactors used in the confirmatory factor analysis. Results showed the CR was higher than 0.70 and the AVE was higher than 0.50, indicating that subfactor reliability was sufficiently high (see Table 3).

#### 3.3.3. Reliability

We tested the reliability of the SOQ’s final version developed in the present study. The Cronbach’s α coefficient of the total 11 questions was 0.867, which indicated high consistency among the items. The Cronbach’s α of the three subfactors ranged from 0.787 to 0.902 (see Table 2).

### 3.4. Convergent and Predictive Validity

We performed a multi-trait/-item matrix analysis to test the questionnaire’s convergent validity. The convergent validity is established when the correlation coefficient calculated after controlling the questions overlapped with the subscales of each question is 0.40 or higher [15]. The analysis showed the correlation coefficients between the individual items and related three subfactors ranged from 0.763 to 0.911, establishing convergent validity. Comparative measures of importance of sexual life to SOQ scores were also significantly associated with the individual 11 SOQ items, three subfactors, and total SOQ scores. The correlation coefficients between the three subfactors with the SOQ’s total score and the importance of sexual life ranged from 0.310 to 0.531 (see Table 4).

### 3.5. The Finalized Model from the Confirmatory Factor Analysis

The following picture describes correlational relationships among three factors and eleven items (see Figure 1; Supplementary material Figure S1). 

### 3.6. Score Differences between Disabled and Non-Disabled

There was a significant mean difference in factors’ and total scores of SOQ between the disabled and non-disabled groups (see Table 5).

### 3.7. Difference between Persons with and without Sexual Problems

There was no mean differences in SOQ factors’ and total scores between the groups with and without sexual problems (see Table 6).

## 4. Discussion

Sexual outlook reflects a person’s psychological state [16] and is expressed differently depending on the individual’s convictions and values, which sociocultural influences affect [17]. The developed questionnaire that has well-validated and reliable items considers South Korea’s cultural characteristics, which are applicable to the general population including people with or without disabilities. We identified the SOQ’s three constructs (personal benefit, relational value, and sexual endeavor) by reviewing previous studies conducted on people with and without disabilities, quantitative and qualitatively.

Most previous studies that conducted research on sexual outlooks in Korea and other countries focused on specific populations such as nurses [18,19], college students [20,21], and elders [5,11]. However, the questionnaire developed in the present study comprehensively embraced a wide range of age groups to measure the general traits of sexual outlooks in adult populations. As sexual outlook is an individual psychological factor [18], it should not be ignored for those with differences such as physical disabilities [22].

Hendrick et al. [4] classified their scale items with four categories: “Permissiveness”, “Birth Control”, “Communion”, and “Instrumentality”. Similar to Hendrick et al., the SOQ developed in the present study has three factors: “personal benefit of sex”, “relational value of sex”, and “sexual endeavor”.

Factor 1 (“personal benefit”) addressed how sex impacted participants’ lives and included three questions about the health-promoting effects of sex in those with and without disabilities who were asked to respond about their recognition of healthiness, youth, and vitality as the benefits of their sex life. Factor 2 (“relational value”) included four questions about sex as an expression of love and a means of communication, the effect on the improvement of the relationship with the spouse (partner), and the willingness to solve sex-related problems. Factor 3 (“sexual endeavor”) included questions about the handling of sex-related problems, consulting with an expert, and sex education. The questionnaire developed in the present study is more convenient than conventional ones and contains essential questions that can induce a positive change in a person’s future sexual life. Therefore, the questionnaire may raise positive sexual interests and considerations among not only the adults who are or were married, but also among elders and people with disabilities who have been neglected and disadvantaged in their sexual lives.

Kedde et al. [23] reported that about 50% of patients who have a disability or chronic disease need professional help in finding sexual partners and adapting to a sex life because of their health issues, and 40% of them need professional help with regard to sexual relations, practical sex-related problems, and sexual impairment. Kedde et al. also reported that two-thirds of participants in their study considered visiting an expert, but only 35% actually visited a medical expert. In addition, only one-third of those who had actually visited an expert responded that they had been positively affected by counseling with the expert. The survey in the present study showed that 35.2% of subjects currently have a sexual disability and the causes of the sexual disabilities were on the order of paralysis (27.8%), decreased sexual desire (25.2%), decreased sexual function (20.9%), and pain during sexual intercourse (13.9%). These results showed the sex-related problems of people with disabilities and suggested that various studies may need to be conducted on participants’ sexual outlooks according to gender, age, and types of disability using a verified scale.

The present study has the following limitations. First, the number of participants with a disability was not sufficiently large; thus, the validity of the questionnaire was not tested as a questionnaire of only people with disabilities. Therefore, a validity test must be conducted with respect to people with disabilities only, after securing a sufficiently large number of samples. Second, because participants in the present study were those residing in Seoul and Gyeonggi-do, the results of the present study may not be generalized to other regions of Korea.

Despite these limitations, the questionnaire developed in the present study was found to be sufficiently reliable. As participants in the present study included men, women, and people with and without disabilities in appropriate ratios, the questionnaire may generally apply to any group of adults, regardless of age and the presence of disabilities in the area of Seoul and Gyeonggi-do. However, the measurement assessed by the SOQ developed in the present study may be applied to capture the current problems of one’s sexual life and sexual outlook so healthcare professionals and heath service researchers could design and employ various sex-related programs to improve their knowledge and quality of life involving sexual health, and resolve current problems through various types of practical studies on sex education.

## 5. Conclusions

The present study was conducted to develop and validate a sexual outlook questionnaire to general populations for use in studies of clinical practice, including sex education and research aimed at improving the quality of human life. The developed questionnaire was designed to examine the sexual outlook of both male and female adults for those married and previously married. The finalized questionnaire includes 11 questions about the three factors of benefit, value, and assertiveness. The SOQ scores range from a minimum of 11 points to a maximum of 55 points, wherein a higher score represents a more positive outlook toward sex. The questionnaire’s reliability and validity were sufficiently tested through several statistical analyses. The questionnaire can be used to conveniently measure the sexual outlooks of any married or previously married adults, regardless of their age group and the presence of disabilities. Sexual education and population-based intervention could be designed based on the needs assessment with this instrument, and sexual health could improve with behavioral change after community intervention.

## 6. Patents

One domestic patent will be registered, and an intellectual property right for the developed questionnaire will be protected in the Republic of Korea.

## Figures and Tables

**Table 1:** General Characteristics of Participants (*N* = 334).

Characteristics	*n* (%)	M ± SD ^1^
Age (year)		48.26 ± 11.09
30–39	78 (23.4)	
40–49	105 (31.4)	
50–49	95 (28.4)	
>60	56 (16.8)	
Sex		
Male	174 (52.1)	
Female	160 (47.9)	
Marital status		
Married	267 (79.9)	
Divorce	33 (9.9)	
Other	34 (10.2)	
Religion		
Yes	206 (61.7)	
No	128 (38.3)	
Education level		
≤Middle school	36 (10.8)	
High school	74 (22.1)	
College or Bachelor	166 (49.7)	
Master or Doctor	58 (17.4)	
Work status		
Employed	232 (69.5)	
Unemployed	102 (30.5)	
Residence		
Seoul	210 (62.9)	
Gyeonggi-do	124 (37.1)	
Chronic disease		
Yes	92 (27.5)	
No	242 (72.5)	
Disability		
Yes	121 (36.2)	
No	213 (63.8)	
Sexual problems		
Yes	115 (34.4)	
No	219 (65.6)	
Importance of sexual life (0 to 10, higher scores mean greater importance)		6.09 ± 2.32

^1^ Note: M = Mean; SD = Standard Deviation.

**Table 2:** Factor Analysis of the 11-Item Sexual Outlook Questionnaire (SOQ) for Adult Populations (*N* = 334).

Items		Factor Loading			Communality	CVI	ITC (r)	Eigenvalues	VE (%)	CV (%)	Cronbach’s α
		Factor 1	Factor 2	Factor 3							
Factor 1 (Benefit)								3.135	28.503	28.503	0.902
x20	Sexual life makes me feel alive.	0.871	0.238	0.105	0.826	0.893	0.899				
x18	Sexual life gives me peace of mind.	0.870	0.238	0.137	0.832	0.857	0.911				
x19	Sexual life increases my self-esteem.	0.813	0.355	0.179	0.819	0.893	0.891				
x17	Sexual life increases the motivation to live.	0.812	0.010	0.206	0.702	0.821	0.827				
Factor 2 (Value)								2.809	25.538	54.042	0.839
x14	Sexual life improves the relationship with the spouse (partner).	0.131	0.846	0.103	0.743	1	0.632				
x11	Sexual life is an expression of love.	0.262	0.785	0.143	0.705	0.964	0.701				
x12	Sexual life is a means of communication.	0.267	0.767	0.059	0.663	1	0.652				
x15	I can discuss a sexual matter with my spouse (partner)	0.055	0.757	0.192	0.613	0.964	0.586				
Factor 3 (Assertiveness)								2.138	19.435	73.477	0.787
x28	If I have a sex problem, I will consult with an expert.	0.078	0.204	0.864	0.794	1	0.677				
x29	If I am given an opportunity, I will receive sex education.	0.115	0.183	0.833	0.740	0.929	0.667				
x27	If needed in my sexual life, I will use a sex aid.	0.347	0.026	0.723	0.644	1	0.593				
Total											0.867
KMO (Kaiser-Meyer-Olkin) = 0.865, Total variance explained = 73.477, x2 = 1996.341 (*p* < 0.001)											

Note: CVI = Content validity index; ITC = Item total correlation; VE = Variance explained; CV = Cumulative variance.

**Table 3:** Validity and Reliability of 11 SOQ Items and Three Factors Through Confirmatory Factor Analysis (*N* = 334).

Item	B	SE	β	CR	Construct Reliability	AVE
Factor 1 (Personal Benefits)					0.92	0.73
x20	1.11	0.08	0.87	14.86 *		
x18	1.12	0.07	0.89	15.12 *		
x19	1.12	0.07	0.90	15.21 *		
x17	1.00		0.70			
Factor 2 (Relational Value)					0.92	0.74
x14	1.33	0.11	0.79	11.81 *		
x11	1.35	0.11	0.81	11.95 *		
x12	1.31	0.12	0.75	11.35 *		
x15	1.00		0.66			
Factor 3 (Sexual Endeavor)					0.78	0.55
x28	1.19	0.11	0.83	10.84 *		
x29	1.13	0.11	0.78	10.78 *		
x27	1.00		0.64			
Model Fitness: χ^2^ = 113.417 *, χ^2^/df = 2.766, RMR = 0.047, RMSEA = 0.073, GFI = 0.941, CFI = 0.963, TLI = 0.950						
Factor 1 & 2 (0.55), Factor 1 & 3 (0.43), Factor 2 & 3 (0.42)						

Note: SE = Standard error; CR = Critical ratio; AVE = Average variance extracted; RMR = Root mean residual; RMSEA = Root mean squared error of approximation; GFI = Goodness of fit index; CFI = Comparative fit index; TLI = Tucker-Lewis index. * *p* < 0.001.

**Table 4:** Correlations of Multi-trait/-item Matrix Analysis for SOQ (*N* = 334).

Factor *n*	Item No.	Factor 1	Factor 2	Factor 3	SOQ_Total
Factor 1: Personal Benefits	x20	0.899	0.428	0.339	0.752
	x18	0.911	0.429	0.354	0.766
	x19	0.891	0.525	0.406	0.811
	x17	0.827	0.249	0.355	0.659
	Factor_1	1	0.456	0.412	0.843
Factor 2: Relational Value	x14	0.339	0.857	0.266	0.593
	x11	0.439	0.844	0.319	0.661
	x12	0.422	0.820	0.257	0.616
	x15	0.285	0.763	0.295	0.541
	Factor_2	0.456	1	0.347	0.735
Factor 3: Sexual Endeavor	x28	0.290	0.330	0.863	0.621
	x29	0.311	0.312	0.844	0.616
	x27	0.430	0.237	0.809	0.636
	Factor_3	0.412	0.347	1	0.745
					
Importance of sexual life	0.526 *	0.374 *	0.310 *	0.531 *	

Note: All correlations of multi-trait/-item analysis were significant at the 0.05 level of *p*-value. * *p* <0.001.

**Table 5:** Differences of 3 Factors’ and Total Scores between Two Groups (*N* = 334).

Factor *n*	Disability		*t* (*p*)
	Yes	No	
	M ± SD ^1^		
Factor 1	13.65 ± 3.21	12.64 ± 3.20	2.763 (0.006)
Factor 2	15.74 ± 2.43	16.14 ± 2.19	−1.530 (0.127)
Factor 3	10.08 ± 2.64	9.12 ± 2.51	3.313 (0.001)
Total	39.48 ± 6.80	37.91 ± 6.03	2.180 (0.030)

^1^ Note: M = Mean; SD = Standard Deviation.

**Table 6:** Differences of 3 Factors’ and Total Scores between Two Groups (*N* = 334).

Factor *n*	Sexual Problems		*t* (*p*)
	Yes	No	
	M ± SD ^1^		
Factor 1	13.24 ± 3.37	12.89 ± 3.17	0.958 (0.339)
Factor 2	15.66 ± 2.47	16.17 ± 2.17	−1.957 (0.051)
Factor 3	9.28 ± 2.76	9.57 ± 2.51	−0.962 (0.337)
Total	38.18 ± 6.87	38.64 ± 6.07	−0.621 (0.535)

^1^ Note: M = Mean; SD = Standard Deviation.

## Supplementary Materials

The following are available online at https://www.mdpi.com/1660-4601/17/22/8681/s1, Figure S1: Figure_SOQ.

## References

[1] Kim S., Kim W., Yoon G., Chae K. (2012). Human Sexuality.

[2] Cuskelly M., Gilmore L. (2007). Attitudes to Sexuality Questionnaire (Individuals with an Intellectual Disability): Scale development and community norms. J. Intellect. Dev. Disabil..

[3] Hudson W.W., Murphy G.J., Nurius P.S. (1983). A short-form scale to measure liberal vs. conservative orientations toward human sexual expression. J. Sex Res..

[4] Hendrick C., Hendrick S.S., Reich D.A. (2006). The brief sexual attitudes scale. J. Sex Res..

[5] Park H., Shin S., Cha H. (2014). Development and Validation of a Sexual Attitude Scale for Elderly Korean People. J. Korean Gerontol. Nurs..

[6] Glasier A., Gülmezoglu A.M., Schmid G.P., Moreno C.G., Van Look P.F. (2006). Sexual and reproductive health: A matter of life and death. Lancet.

[7] Inelmen E.M., Sergi G., Girardi A., Coin A., Toffanello E.D., Cardin F., Manzato E. (2012). The importance of sexual health in the elderly: Breaking down barriers and taboos. Aging Clin. Exp. Res..

[8] Degauquier C., Absil A.-S., Psalti I., Meuris S., Jurysta F. (2012). Impact of aging on sexuality. Rev. Med. Brux..

[9] Derogatis L.R. (1980). Psychological Assessment of Psychosexual Functioning. Psychiatr. Clin. N. Am..

[10] Lee Y. (2012). The Differences of Sexual Attitudes and Sexual Behaviors Based on the Married Women’s Type of Orgasm and Orgasmic Disorder. Korean J. Woman Psychol..

[11] White C.B. (1982). A scale for the assessment of attitudes and knowledge regarding sexuality in the aged. Arch. Sex. Behav..

[12] Tabachnick B.G., Fidell L.S., Ullman J.B. (2007). Using Multivariate Statistics.

[13] Hair J.F., Black W.C., Babin B.J., Anderson R.E. (2006). Multivariate Data Analysis.

[14] Yu J.P. (2016). Concept and Understanding of Structural Equation Model.

[15] Ware J.E., Snow K.K., Kosinski M., Gandek B., The Health Institute, New England Medical Center (1993). SF-36 Health Manual and Interpretation Guide.

[16] Ayalon L., Gewirtz-Meydan A., Levkovich I. (2019). Older Adults’ Coping Strategies with Changes in Sexual Functioning: Results from Qualitative Research. J. Sex. Med..

[17] Abdolmanafi A., Nobre P., Winter S., Tilley P.M., Jahromi R.G. (2018). Culture and Sexuality: Cognitive–Emotional Determinants of Sexual Dissatisfaction Among Iranian and New Zealand Women. J. Sex. Med..

[18] Reynolds K.E., Magnan M.A. (2005). Nursing Attitudes and Beliefs toward Human Sexuality: Collaborative research promoting evidence-based practice. Clin. Nurse Spec..

[19] Kim H.W., Jung Y.Y., Park S. (2012). Evaluation and Application of the Korean Version of the Sexuality Attitudes and Beliefs Survey for Nurses. J. Korean Acad. Nurs..

[20] Nemcić N., Novak S., Marić L., Novosel I., Kronja O., Hren D., Marušić A., Marušić M. (2005). Development and validation of questionnaire measuring attitudes towards sexual health among university students. Croat. Med. J..

[21] Sung K.M., Lee S.Y. (2018). Development of sexual values scale for college students. J. Korean Soc. Sch. Health.

[22] Kim S.H., Lee B.S., Han S.J. (2014). Sexual activity and factors influencing the sexual adjustment in men with spinal cord injury. J. Korean Clin. Nurs. Res..

[23] Kedde H., Van De Wiel H., Schultz W.W., Vanwesenbeeck I., Bender J. (2012). Sexual Health Problems and Associated Help-Seeking Behavior of People With Physical Disabilities and Chronic Diseases. J. Sex Marital. Ther..

