# Supplementary figures and images for: Development and Validation of a Sexual-Outlook Questionnaire (SOQ) for Adult Populations in the Republic of Korea
Source: Int J Environ Res Public Health. 2020 Nov 23;17(22):8681. doi: 10.3390/ijerph17228681 (PMC7700134; doi:10.3390/ijerph17228681)

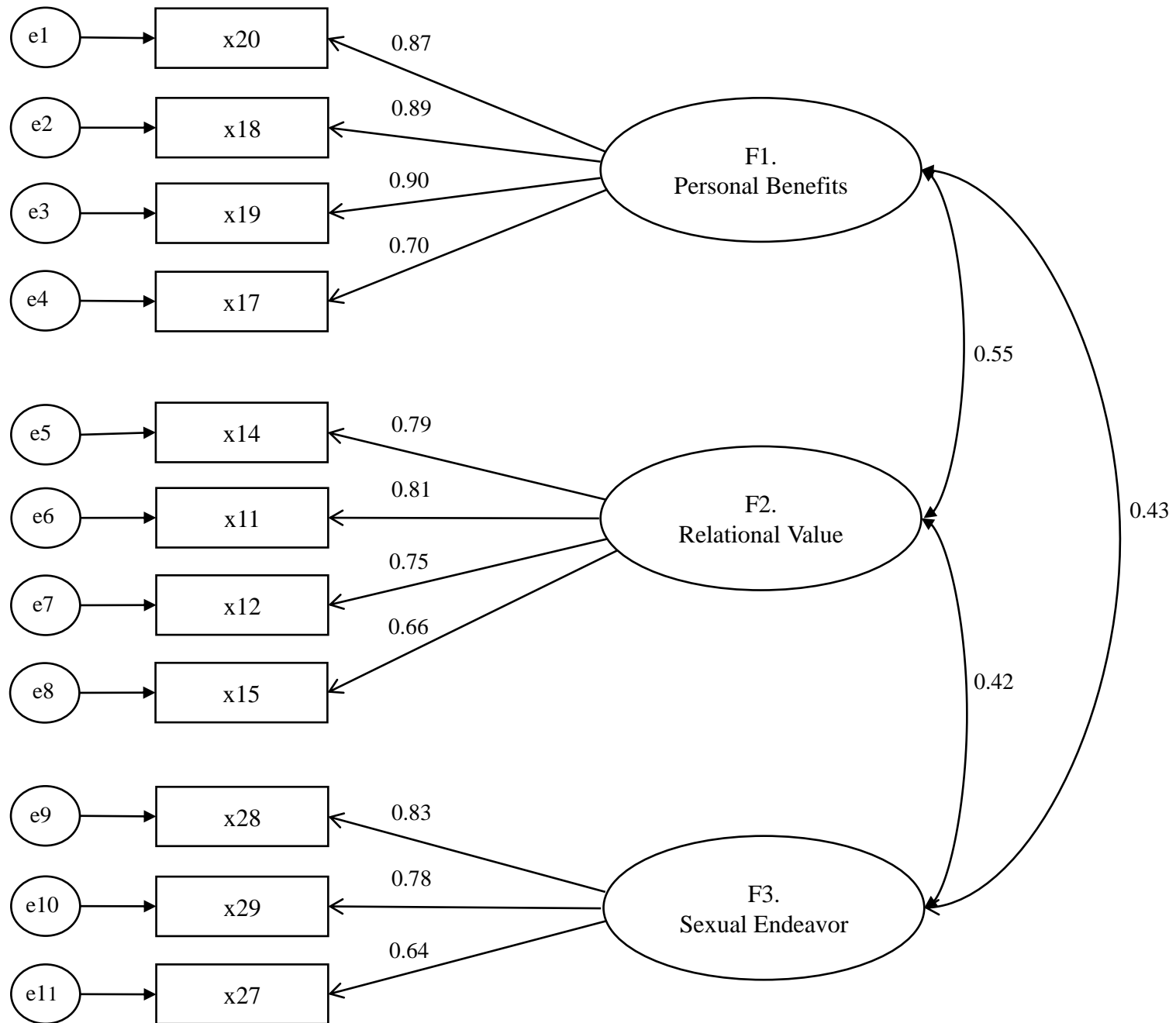

Supplement: Supplementary file 1 [file ijerph-17-08681-s001.zip › ijerph-985578Figure_SOQ.pdf]
